# Enhancing skin tone representation in optical vascular phantoms

**DOI:** 10.1117/1.JBO.31.7.075001

**Published:** 2026-07-22

**Authors:** Anni Ranta-Lassila, Lauri Rannaste, Jarno Petäjä, Marko Korkalainen, Markku Alamäki, Alexey Popov

**Affiliations:** VTT Technical Research Centre of Finland, Oulu, Finland

**Keywords:** optical phantom, vascular phantom, microfluidics, vasculature, skin tone, optical properties, mechanical properties, spectroscopy, hyperspectral imaging

## Abstract

**Significance:**

Racial disparities and bias in the performance of optical medical devices, particularly in individuals with darker skin tones, have raised concerns about diagnostic accuracy and healthcare equity. To address the issue of misdiagnosis in people of color, there is a critical need for more representative tools in device testing and development. Skin-mimicking optical phantoms spanning a broad range of skin tones provide a practical solution, enabling more equitable and accurate evaluation of optical technologies.

**Aim:**

We aimed to develop optical skin-mimicking phantoms representing a range of skin tones for use in the development and evaluation of optical medical and consumer wearable devices.

**Approach:**

Multi-layered water-free silicone-based optical phantoms were designed and fabricated to replicate the optical and mechanical properties of the skin. A vascular structure was integrated into the phantoms, around which a micropump system was developed to simulate blood flow. The phantoms were evaluated using spectroscopy and hyperspectral imaging.

**Results:**

The phantoms of varying skin tones with corresponding individual typology angle values spanning from 51.4 to −7.5  deg exhibited optical and mechanical properties comparable to real skin reflectance spectra across different pigmentation levels. Closer spectral agreement with skin was achieved using coffee compared with commercial pigment mixtures. The spectral signature of the blood-mimicking liquid was effectively masked by the darker superficial phantom layer mimicking heavily pigmented epidermis.

**Conclusions:**

Optical skin-mimicking phantoms containing vascular structures with various skin tones were demonstrated, showcasing their potential as valuable and durable tools for improving the development of optical medical devices and consumer wearables for diverse populations.

## 1 Introduction

Understanding light interaction with biological tissues is crucial for the advancement in optical diagnostics, therapy, medical imaging, and cosmetics applications. For these technologies, optical tissue-mimicking phantoms provide a controlled, stable, reproducible, available around the clock and cost-efficient environment allowing for more efficient and reliable testing compared with real skin.^1^^,^^2^ Liquid,^3^^,^^4^ gel/agar/gelatine-based,^5^ and solid^6^ phantoms are routinely used in academic research. Liquid phantoms made from Intralipid or polystyrene beads suspended in water used as scatterers with the addition of artificial or natural absorbers (e.g., Indian ink, nigrosine, whole blood/washed red blood cells/hemoglobin) to mimic single or broadband spectral signatures of biotissue offer fast fabrication and highly reproducible samples.^7^ However, they often struggle to address different shapes and sizes, incorporation of self-suspended vessels (capillary networks), and stability/usability over extended periods, especially at room temperature. Gel/agar/gelatine phantoms partially solve this problem, but their fragility, reliance on unstable organic components,^8^ inability to implement millimeter-scale dimensions, and incorporation of tubeless (micro)capillaries make them a non-ideal solution. Solid phantoms can overcome all of the mentioned limitations and are generally perceived as the most desirable type of phantoms suitable for long-term (months and years) use in different locations and across different instruments. They are based on a mixture of scattering powders (${\mathrm{TiO}}_{2}$ and ZnO) and liquid or powder absorbers embedded into a matrix, which is often silicone-based.^9^^,^^10^ To tackle powder inhomogeneous distribution and sedimentation caused by improper mixing, a particle-free solution was proposed.^11^ Rapid advancement of three-dimensional (3D) printing technology, from plastic filament melting^12^ to photopolymerization of resins by LEDs or lasers,^13^ has resulted in affordable products and their wide use, although tuning of mechanical properties and implementation of microfeatures require further development of printing materials (resins).^14^

Solid phantoms with a microfluidic network mimicking skin morphology and reflectance in the visible near-infrared spectral range are a scarce resource, even in academic settings due to the multidisciplinarity of the topic and a variety of expertise, technologies, and equipment required for their implementation. Over recent years, attempts were made to cover skin tones and capillary structure individually. Synthetic melanin,^15^ coffee,^16^ and a combination of synthetic dyes^17^ were used for spectral engineering and a variety of approaches for vessels fabrication—from the use of solid^18^ and liquid^19^ metal structures with subsequent solidification and further etching or pulling them out^20^ to 3D printing^21^ and nature-inspired porous structures.^22^ Efforts for implementing a pulsatile flow deserve their own description.^23^^,^^24^ However, merging of the broadband optical and microfluidic domains is very limited and is represented by relatively simplistic structures.

The challenges in fabricating skin-mimicking phantoms are associated with the implementation of the spectral properties closely reproducing those of skin layers and the skin as an integral object and a desire to avoid the use of natural materials such as melanin, oxy- and deoxy-hemoglobin, and water. Another challenge is to address geometrical constraints such as the thickness of the epidermis (100  μm or smaller), sizes of the blood vessels including capillaries (from units of μm to hundreds of μm) and their embedding depth (100  μm and lower), and arrangement of realistic blood flow. The materials used in skin phantoms are by large the same as for solid tissue phantoms (matrix, scatterers, and absorbers).^25^ Small thickness of the epidermal layer is addressed by the spin-coating fabrication method.^26^^,^^27^ Melanin substitute in the form of polydopamine^28^ shows recent promise not only in the visible near-infrared range but up to 15-μm wavelength. Capillary network implementation is very often tackled by embedding metal wires with diameters of tens of micrometers^29^ into the mold and subsequent pulling them out after phantom solidification or by embedding mm and sub-millimeter tubing.^30^ Very recently, new data-driven approaches combined with 3D printing and molding have emerged,^31^ although with transparent materials. Due to the 50th anniversary of the invention of pulse oximetry in 2024, several papers were published to address the effect of skin tone on the photoplethysmography (PPG) signal^32^^,^^33^ but without comparison of the phantom reflectance to real skin and using relatively bulky pumping systems for creating a pulsatile flow. Useful approaches of phantom implementation have been developed in complementary application fields of biomedical optics, such as optical coherence tomography and digital holographic microscopy,^34^ but uptake and knowledge transfer across different disciplines remain extremely limited.

As a result, limited diversity in skin tone representation within phantoms can lead to inaccuracies in device performance when applied to individuals with darker skin tones and further to misdiagnoses or suboptimal treatments. Racial disparities have been reported in the performance of various optical diagnostic devices, including wearable sensors, and pulse and cerebral oximeters, especially during the COVID-19 pandemic.^35^^–^^38^ This discrepancy highlights the need for phantoms that mimic a wide range of pigmentation levels, ensuring that optical techniques are equitable and accurate for diverse populations.

To address this issue, we developed multi-layered phantoms mimicking different skin tones with individual typology angle (ITA) values ranging from 51.4 to −7.5  deg. Within these phantoms, we integrated a vascular-mimicking microstructure that demonstrates the potential applicability of these phantoms in blood-flow-related health monitoring. The effect of the melanin-mimicking layer on the imaging of the integrated vasculature was demonstrated.

## 2 Materials and Methods

### 2.1 Single-Layer Optical Phantoms

To accurately replicate optical properties of the human skin, phantoms should possess relevant absorption and scattering characteristics. Silicone (Ecoflex 00-30, Smooth-On, Inc., Macungie, Pennsylvania, United States, and Sylgard 184, Dow, Midland, Michigan, United States) was chosen as a base material due to its skin-like mechanical properties, durability, and ease of processing. Zinc oxide (ZnO; Zinc oxide 205532, Sigma-Aldrich, St. Louis, Missouri, United States) powder was used as a scattering agent, selected for its stability, cost-effectiveness, and high refractive index. Commercial skin-shade silicone pigments (Silc Pig, Smooth-On, Inc., and SilTone, FormX, Sunnyvale, California, United States) and coffee (decaff instant coffee, Douwe Egberts, Amsterdam, The Netherlands) were used as absorbers. Silicone pigments offer long-term stability, compatibility with silicone matrices, and customizable color mixing. Coffee exhibits a melanin-like absorption spectrum in the visible-near infrared spectral range,^39^ making it a more biologically relevant absorber for mimicking optical properties of skin. Although synthetic dyes, such as nigrosin, provide a closer spectral match to melanin for a wavelength of >600  nm,^40^ absorption characteristics of coffee are sufficient for reproducing physiologically relevant bulk absorption behavior required for the skin phantom. Moreover, in the blue-green range (<600  nm) where skin tone has a higher impact on optical measurements, in contrast to nigrosine, coffee is a well-suited substitute for melanin.^39^ In addition, coffee is inexpensive and widely available, which improves experimental reproducibility and repeated phantom fabrication without the need for chemical processing.

For the realization of scattering homogeneous phantoms and skin-mimicking layers, ZnO powder was added into the silicone base to achieve ZnO concentrations ranging from 0 to 25  mg/g. The mixtures were thoroughly blended using a speed mixer (SpeedMixer™, Hauschild, Hamm, Germany) for 2.5 min at 2500 rpm and poured into purposely fabricated plain metallic molds. The mixtures within the molds were then degassed in a desiccator, with air removed with a pump (ILMVAC GmbH, LK 131 Z, Ilmenau, Germany), and finally cured in an oven (Memmert, UFE 400, Schwabach, Germany) at 70°C for 10 min.^41^

Properties of the thin (ca. 1 mm) ZnO-containing phantom layers, such as transmittance and reflectance, refractive index, and thickness values, were measured with a spectrophotometer with an integrating sphere (Cary 5000 with white standard included, Agilent Technologies, Santa Clara, California, United States), a refractometer (DR-M2/1550, Atago, Tokyo, Japan), and an optical microscope (ECLIPSE LV150N, Nikon, Tokyo, Japan) with an optical coherence tomograph (OQ Labscope 3.0, Lumedica, Durham, North Carolina, United States), respectively. These values were then used as an input into an iterative inverse adding–doubling (IAD) freeware^42^ to recover their absorption and scattering properties. The IAD program iteratively estimates optical parameters (absorption and reduced scattering coefficients) of a thin slab by forward-modeling reflectance and transmittance using the adding–doubling method. At each iteration, the computed reflectance and transmittance are compared against the experimentally measured values, and the optical parameters are updated until convergence.

For the coffee layers (representing epidermis), instant coffee powder was diluted in deionized water to form a 0.5  g/mL concentration. This suspension was further added to the silicone base to form 2, 4, and 8  mg/g coffee–silicone concentrations. The mixtures were then blended, degassed, molded, and cured, as described above. The cured phantom layers were again measured for their optical and physical properties (as above), further used to recover the optical parameters (absorption and reduced scattering coefficients).

Based on the measurements of these initial phantoms, appropriate concentrations for the scattering and absorbing additives were calculated to replicate the optical characteristics of skin.

For fabricating homogeneous (single layer) slabs composed of skin-tone silicone pigments, color pigments were mixed with the silicone base using 4  mg/g of pigment and 5.7  mg/g of ZnO. “Light” pigment was used for the light-tone phantom; “medium” pigment, for the medium tone; 3:1 “medium”: “black” pigment mixture, for the medium dark; and 1:1 “medium”: “black” pigment mixture, for the dark tone phantoms.

### 2.2 Vascular Structure Molds

The dermis contains a highly structured vascular network with capillaries appearing at multiple depths, with the highest density in the papillary dermis.^43^^,^^44^ Vessel diameters range from 4-μm capillaries to 300-μm hypodermal arteries and 1000-μm veins at the dermal–subcutaneous boundary.^45^^,^^46^ More details on human vascular structure can be found in Sec. S1 in the Supplementary Material. The vessels demonstrated in this work do not represent the whole dermal vascular network, as they differ in size and shape at different locations of the human body. The implemented vessels were inspired by the dense vascular network in the hand area where wearables are commonly located.

**Fig. 1:**
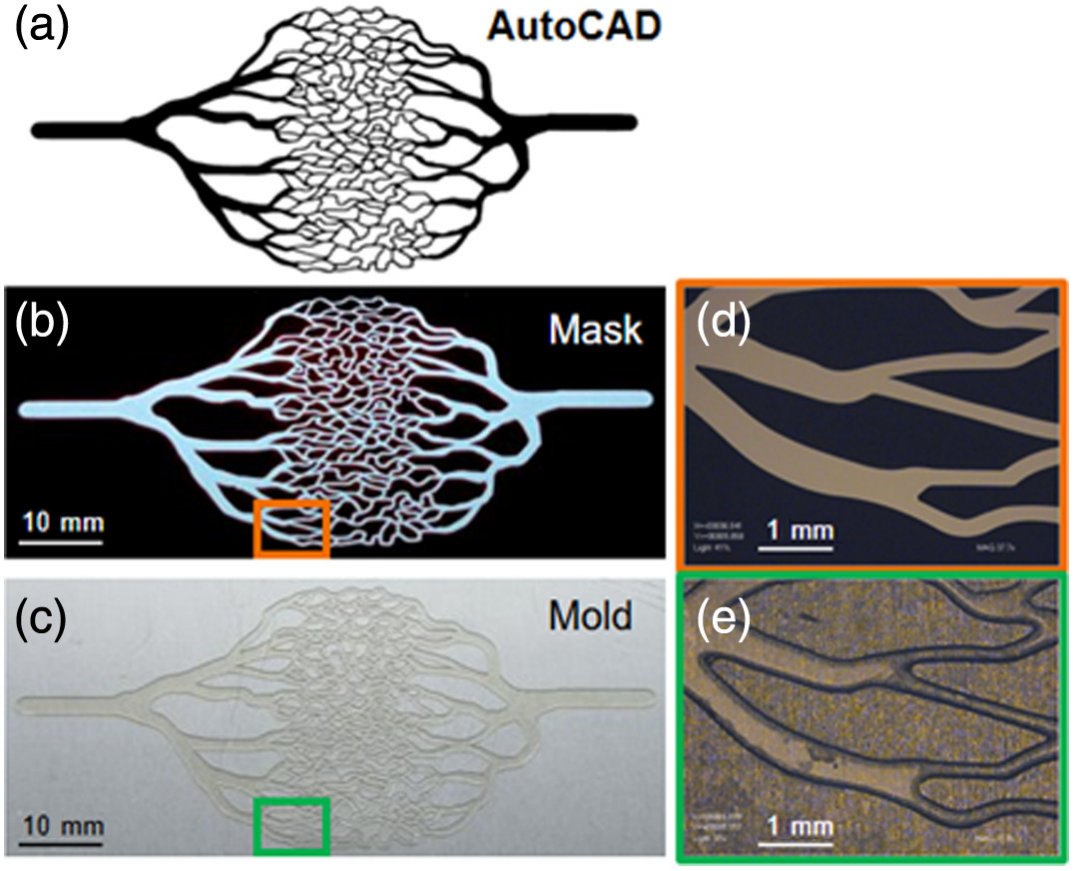
Vascular structure. (a) Mask design. (b) Mask implementation. (c) Vascular structure mold. (d) and (e) Optical microscopy images of the same location of the mask and mold, respectively.

We used the methodology of fabricating a vascular structure previously described in Ref. 47, with modifications. The vascular structure was designed in AutoCAD 2024 (Autodesk, San Francisco, United States) and used for the manufacturing of a soda–lime glass photolithography mask (JD Photo Data, Hitchin, United Kingdom). The mask was then used for fabricating vascular structure molds with ultraviolet backside lithography (Mask Aligner MA6, Suss MicroTech, Garching, Germany) on the 200-μm-thick dry film photoresist. A detailed description of the backside lithography process is described in Sec. S2 in the Supplementary Material. We fabricated various-sized single- and multi-layered vascular phantoms with integrated vascular networks. Figure 1 presents the design, mask, and fabricated mold of the vascular structure.

**Fig. 2:**
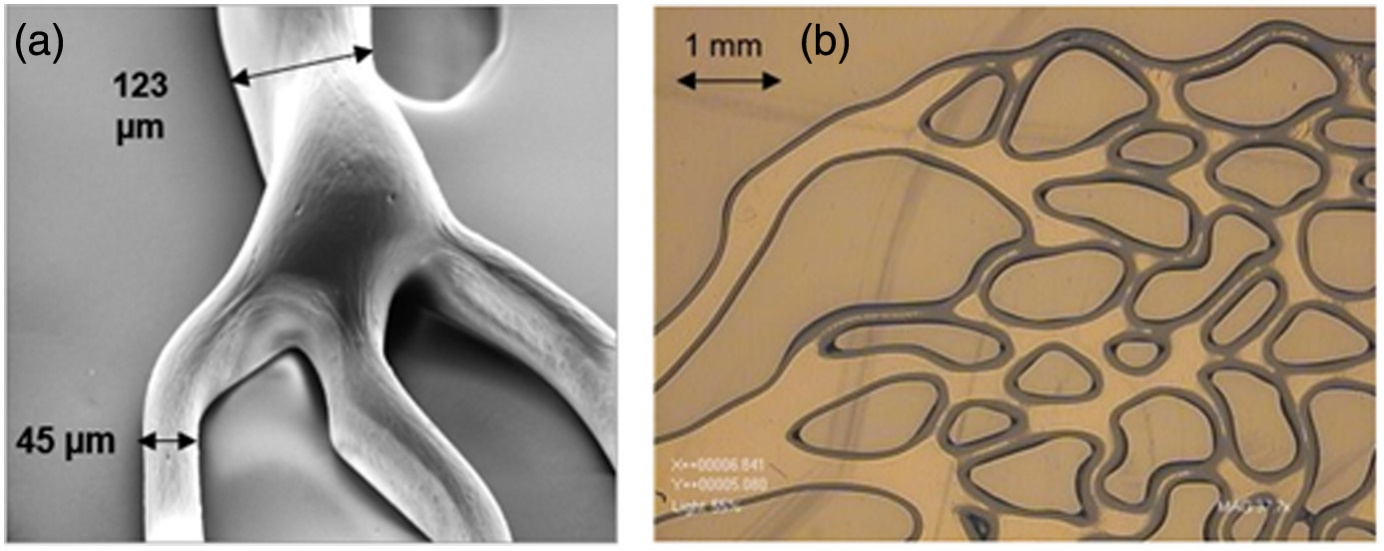
Vessel structure of the vascular mold. (a) SEM imaging. (b) Optical microscopy with episcopic illumination.

The vascular patterns were imaged with scanning electron microscopy (SEM) (NeoScope JCM-5000, JEOL, Tokyo, Japan) and optical microscopy (SmartScope ZIP250, OGP, Rochester, New York, United States) (Fig. 2).

The rounded shape of the capillary mold is clearly visible [Fig. 2(a)]. However, the pattern clearly shows that the wide capillaries have a flat top surface [Fig. 2(b)] when imaged with an optical microscope and episcopic illumination. This indicates that the wider capillaries would benefit from a thicker dry film to achieve rounded shapes. After fabrication and testing of several sizes of vascular patterns, a structure with a height of 0.2 mm, a width of 32.5 mm, a length of 74.5 mm, and capillary lateral sizes ranging from 0.2 to 2 mm was used for phantom implementation.

### 2.3 Multi-Layered Phantoms

**Fig. 3:**
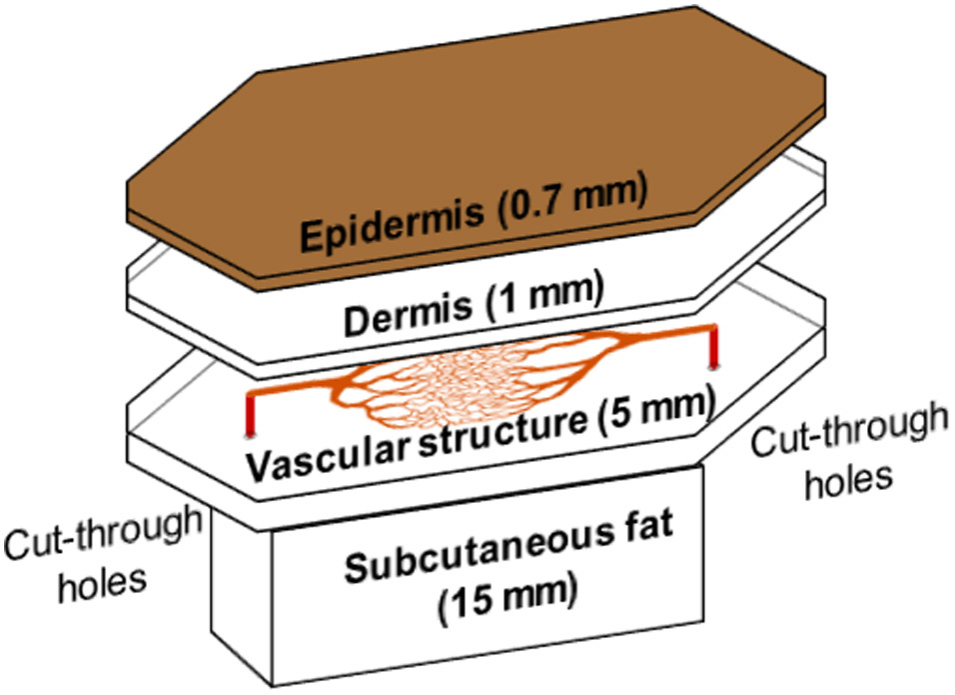
Schematic of the multi-layered phantom, with the numbers indicating the thickness of the constituting layers. The height of the vascular structure is ca. 200  μm, but the layer hosting it is 5 mm thick and represents the upper part of the subcutaneous layer (with the overall thickness 15+5  mm).

For improved skin emulation, multi-layered phantoms were designed and fabricated to mimic the three main layers of skin (top to bottom): epidermis, dermis with an integrated vascular structure, and subcutaneous fat (Fig. 3). The thicknesses of the layers were set to 0.7, 1, and 20 mm, respectively, as a compromise between the fabrication constraints and the goal of reproducing realistic overall optical properties of the human skin. Although the epidermal thickness *in vivo* is typically <0.1  mm,^3^ our primary objective was not to exactly match anatomical thickness but to ensure that the reflectance spectrum of the multi-layered phantom resembles that of real skin. This aspect is largely absent in existing literature, which often focuses on individual layer properties rather than on the optical behavior of the skin as a whole.

Silicone mixtures containing varying amounts of additives (ZnO and coffee) were poured into plain metallic molds to realize layers of these thicknesses and were cured at 70°C for 1 h in the oven. The mold used for the 5-mm upper part of the subcutaneous fat layer included the vascular structure.

The dermal and fat layers contained 5 and 3.75  mg/g of ZnO, respectively, while the epidermal layers (of different color tones) contained both 7.5  mg/g of ZnO and varying concentrations (2, 4, and 8  mg/g) of coffee. Such concentrations aimed to mimic reflectance spectra of light (European), medium (South Asian), and dark (African) skin.^48^ The fat layer was bonded to the dermal layer after oxygen plasma treatment (TePla 440G, TePla Technics, Wettenberg, Germany, 300 W, 30 s), leaving the (tubeless) vascular structure in between. The epidermal layers (of different coffee content) were kept unbonded on top of the dermal layer for easy skin tone alteration during measurements. The intrinsic surface adhesion of poly(dimethylsiloxane) (PDMS) ensured close contact of the epidermis with the underlying layers, preventing air gaps. Refractive index difference among the layers is ca. 0.001, minimizing undesired intralayer reflectance.

### 2.4 Micropump System

**Fig. 4:**
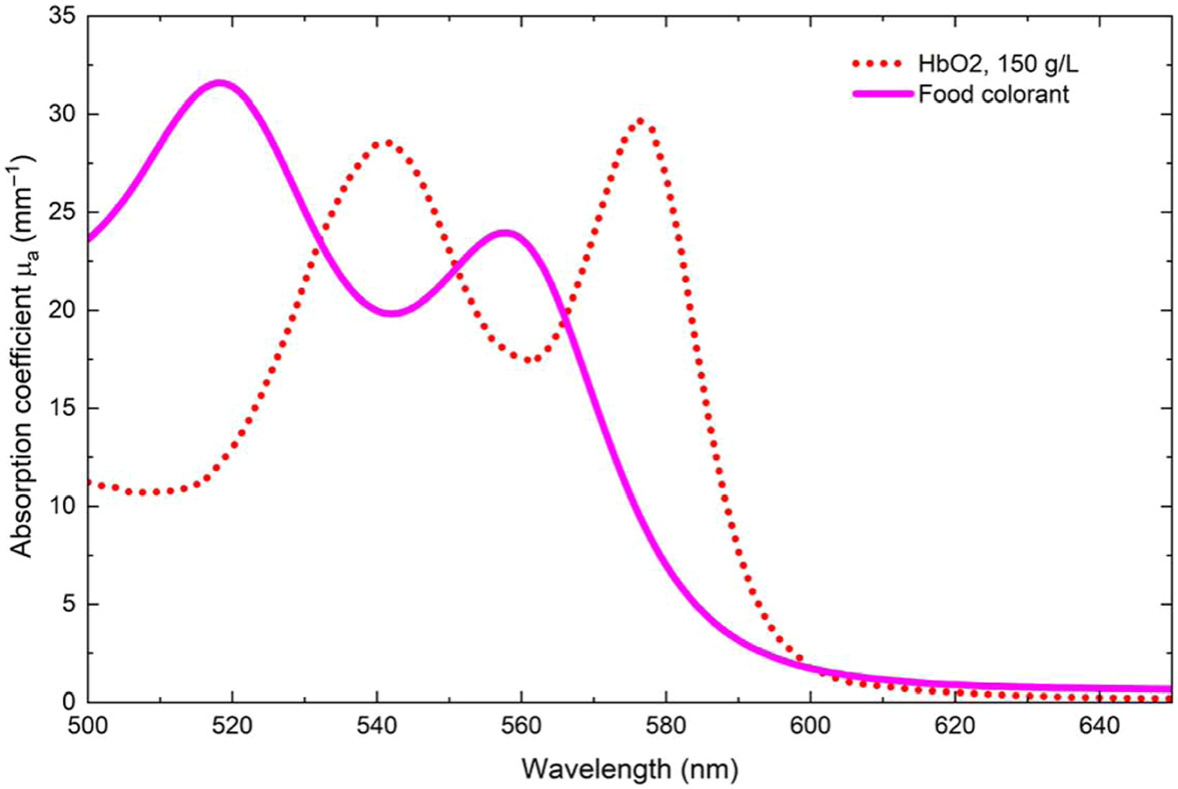
Absorption spectra of food colorant (50%) and oxygenated hemoglobin (150  g/L, corresponding to whole blood).

To enable blood-mimicking liquid flow through the phantoms, a piezoelectric double diaphragm pump (mp6-liq, Bartels Mikrotechnik, Dortmund, Germany) was connected via a 1-mm inner diameter silicone tubing (mp-s, Bartels Mikrotechnik) and 0.61-mm outer diameter 90-deg angled dispensing tips (Adhesive Dispensing Solutions, Buckinghamshire, United Kingdom). The system was controlled by an in-house developed Qt-based software and utilized an mp-Multiboard2 microcontroller (ESP32, Bartels Mikrotechnik) along with $I^{2}C$ drivers to adjust frequency and voltage of the pumps. A pressure sensor by Honeywell was installed close to the micropump outlet for monitoring purposes. Both steady (non-pulsatile) and pulsatile user-defined flows were implemented to extend the applicability of the phantoms. Although hyperspectral imaging (HSI) experiments require only stationary liquid flow, pulsatile operation enables the use of the same phantoms for PPG sensing. Red food colorant (Red, Dr. Oetker, Bielefeld, Germany) was used to mimic human blood due to the proximity of its absorption spectrum to that of oxyhemoglobin (Fig. 4)^49^^,^^50^ and ease of handling without the need for an ethical permit.

**Fig. 5:**
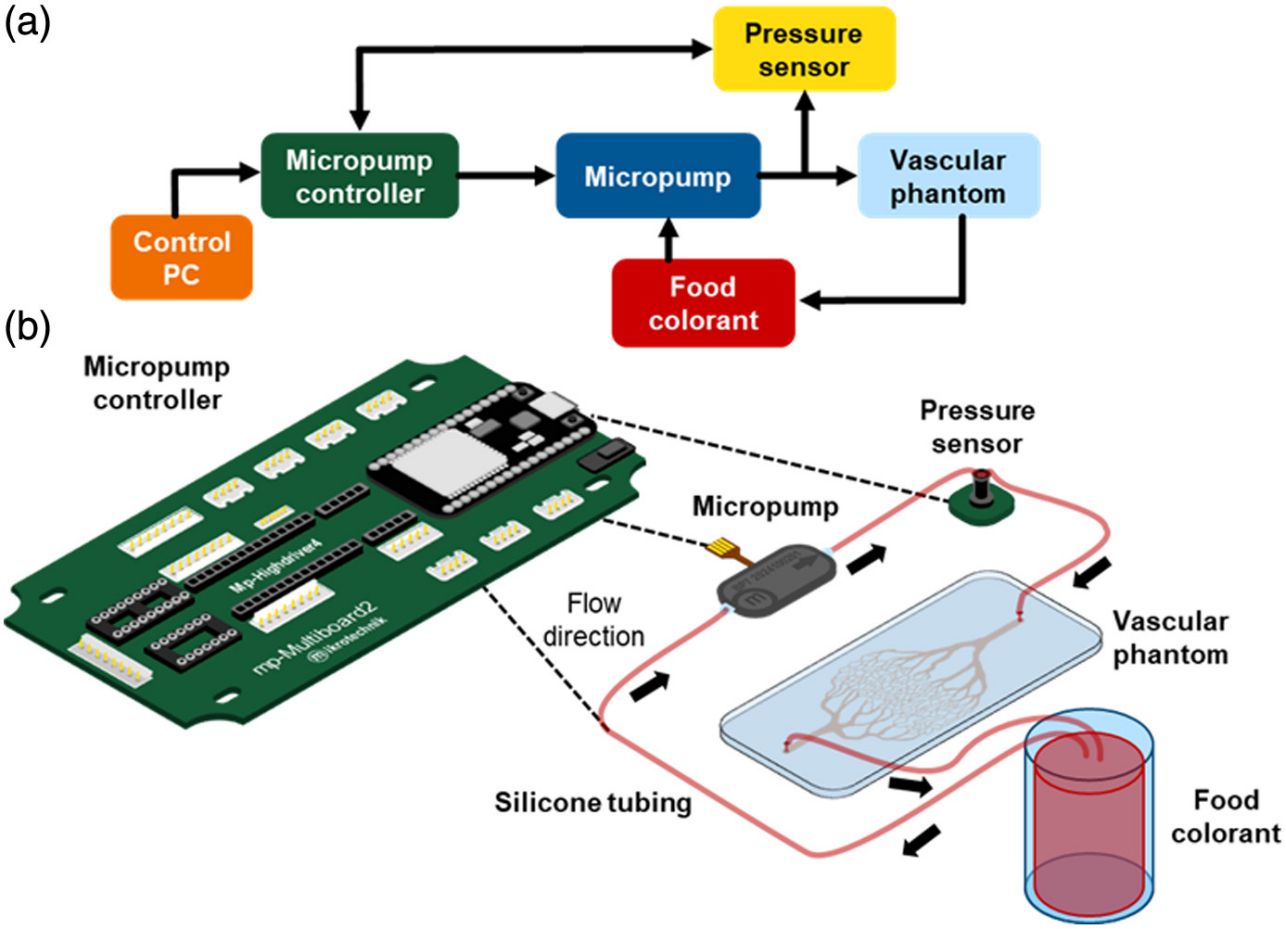
Artificial cardiovascular system. (a) Schematic and (b) graphical illustration of the micropump system for perfusing blood-mimicking fluid through the skin-mimicking phantoms. The transparent phantom is depicted for illustrative purposes. In experiments, the multi-layered phantom with different skin tones was used.

Figure 5 shows the micropump (artificial cardiovascular) system perfusing blood-mimicking fluid through the vascular phantom.

### 2.5 Hyperspectral Imaging

**Fig. 6:**
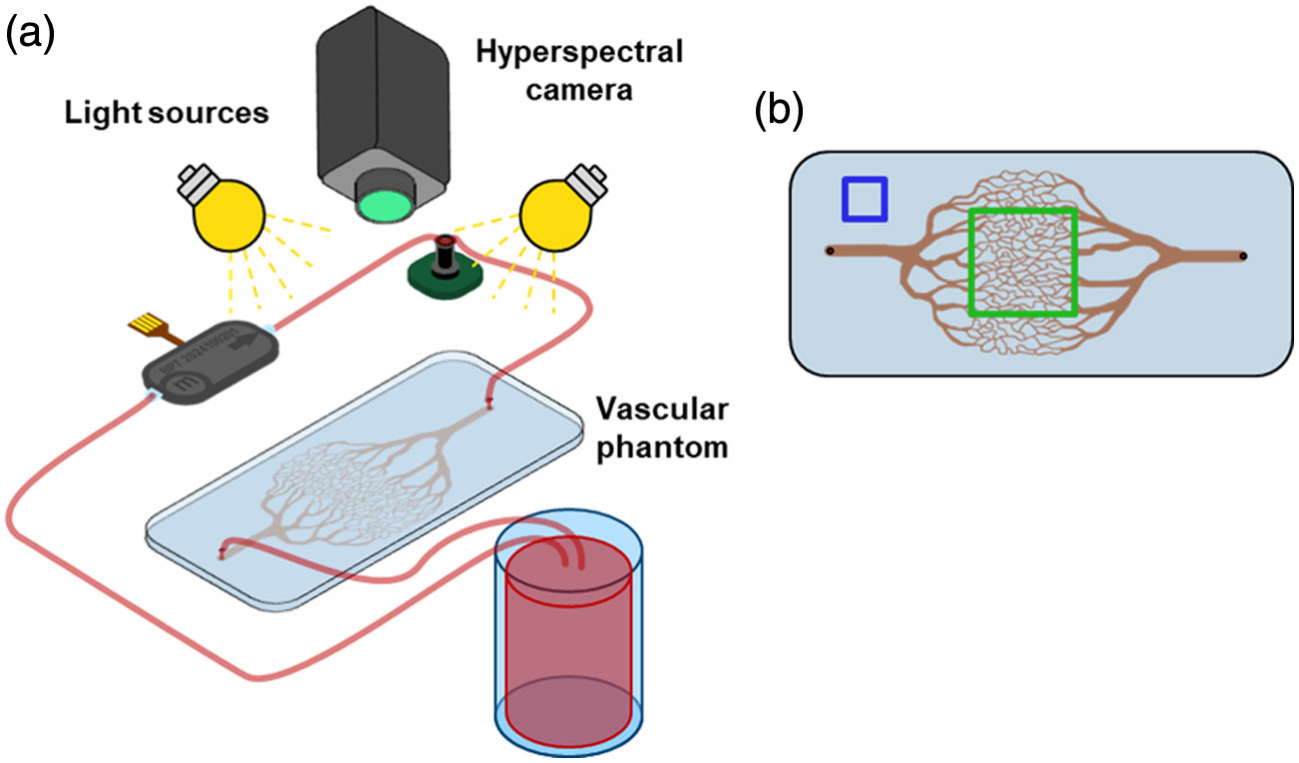
(a) Hyperspectral imaging setup (with micropump controller omitted). (b) ROIs of the phantom. Two ROIs (denoted by the large green and the small blue rectangles) were selected, corresponding to the areas with and without vascular structure. The transparent phantom is depicted for illustrative purposes. In experiments, the multi-layered phantom with different skin tones was used.

Imaging with a hyperspectral camera [Specim IQ, Specim (white standard included), Oulu, Finland] was performed to evaluate the effect of the phantoms with varying skin tones on the detectability of blood-mimicking liquid. Incandescent lamps were used as broadband light sources. Prior to the experiments, the camera was calibrated using a complementary white standard (Specim) following the procedure prescribed by the manufacturer. The measured data (hypercubes) were stored on the camera memory card and used for further processing. The multi-layered phantoms with coffee-containing layers of varying darkness were imaged with and without blood-mimicking liquid flow enabled by the micropump system. From the acquired images, square-shaped regions of interest (ROIs) around the vessels and in the surrounding area were selected. Each ROI selection was made as large as possible without including unwanted features. The average reflectance values were calculated for each area and wavelength. Figure 6 shows a schematic of the imaging setup and the selection of the ROIs.

## 3 Results

### 3.1 Skin-Toned Optical Phantoms

**Fig. 7:**
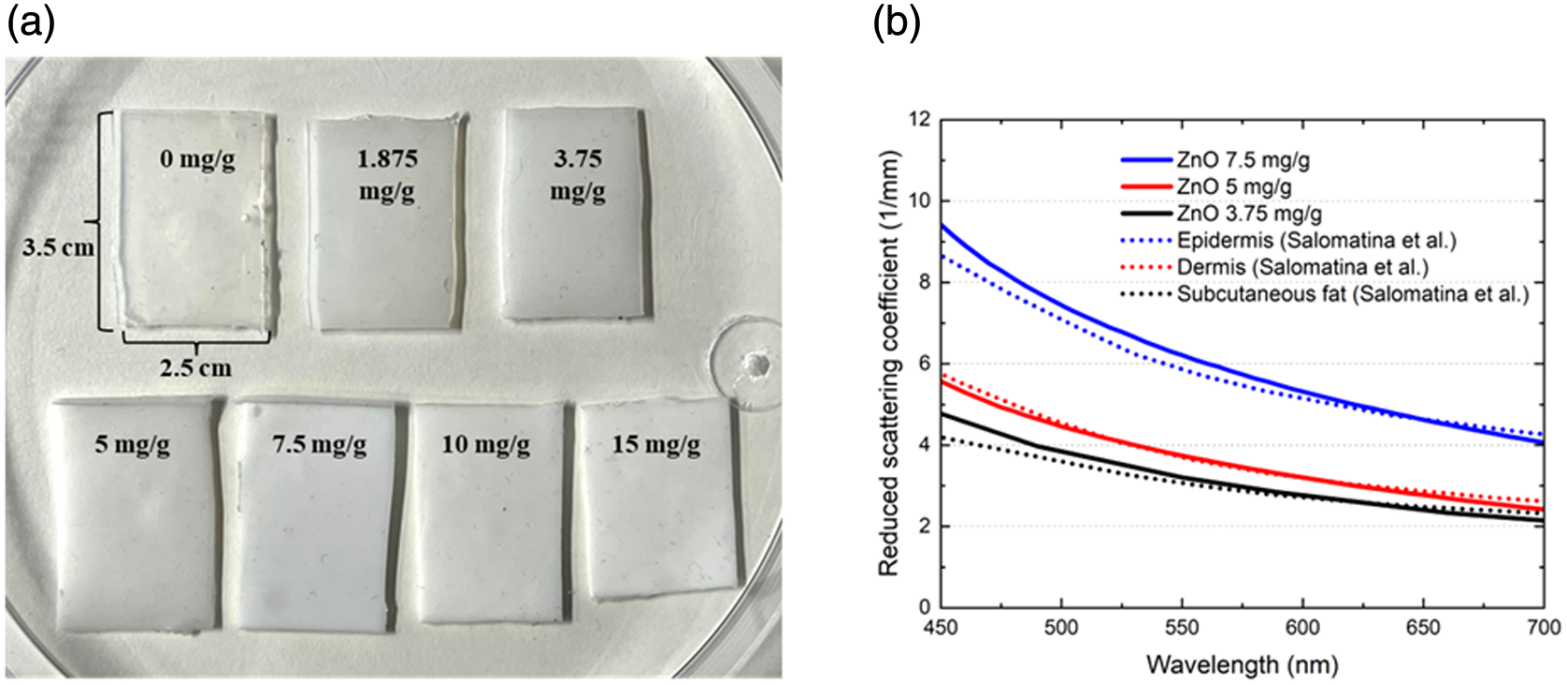
(a) Photos of 1-mm single-layer silicone phantoms with varying ZnO concentrations (0 to 25  mg/g) in a Petri dish. (b) Reduced scattering coefficients of these layers (reference values for the skin layers are from Ref. 51).

Initially, thin (ca. 1 mm) phantom layers were fabricated to reveal optimal concentrations of scattering and absorbing agents (ZnO, commercial color pigments, and coffee). The scattering of the thin phantoms was compared with experimental values of different skin layers, reported in Salomatina et al.,^51^ for Caucasian skin. The phantoms, their scattering coefficients, and the reference values are presented in Fig. 7.

**Fig. 8:**
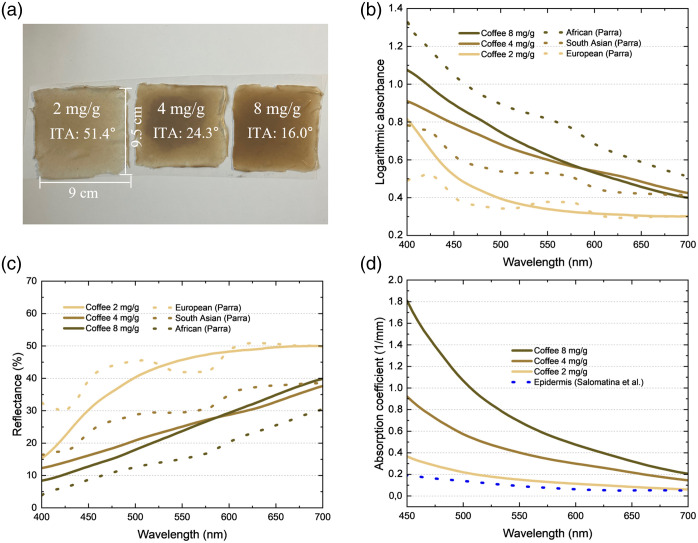
(a) 0.7-mm-thick coffee-containing layers with 2, 4, and 8  mg/g coffee concentrations, (b) absorbance, and (c) reflectance of an assembly (each coffee-containing layer on top of a 20-mm-thick scattering ZnO-containing multi-layered phantom) compared with real skin from Ref. 48. (d) Absorption coefficients of individual coffee phantom layers compared with reference epidermal layer of Caucasian skin from Ref. 51.

From the comparison [Fig. 7(b)], appropriate ZnO concentrations were recovered for different skin-mimicking phantom layers: 7.5, 5, and 3.75  mg/g for the epidermis, dermis, and subcuteneous fat, respectively. For finding correct absorption properties, phantoms containing silicone color pigments and coffee were compared with real skin absorbance, reported by Parra.^48^ Logarithmic absorbance A was calculated from the reflectance and transmittance measurements, using the equation $A=-\log_{10}(R+T)$, where R is the reflectance and T is the transmittance of the phantom. For comparison, thick (ca. 2 cm) single-layer homogeneous phantoms containing mixtures of silicone pigments and multi-layered phantoms comprising thin (ca. 1 mm) coffee-bearing layers on top of 20-mm ZnO-containing stacks were used. From the comparison, appropriate concentrations were calculated. Based on these, tissue-mimicking phantoms possessing both scattering and absorbing properties were fabricated. The absorbance and reflectance spectra of the silicone pigment phantoms showed moderate agreement with the corresponding skin tones reported in Ref. 48, while the spectral shapes of the coffee-based phantoms more closely resembled those of real human skin. Therefore, the work continued with the coffee-containing phantoms. Figure 8 presents the epidermal coffee phantom layers, measured absorbance and reflectance on top of the 20-mm ZnO-containing multi-layered phantom, and comparison with skin data from Ref. 48. Absorption coefficient is also presented and compared with reference value from Ref. 51. Figures of the silicone pigment phantoms can be found in Sec. S3 and Fig. S4 in the Supplementary Material.

Figure 8(a) presents photos of the thin epidermis-mimicking coffee-containing layers. Despite adding the coffee incrementally and using a speed mixer for several minutes, some local inhomogeneities in coffee distribution were observed in certain phantoms. This is likely due to the clustering of the particles during curing. Although natural skin exhibits local variations in pigmentation, for applications requiring highly reproducible skin-tone phantoms, further optimization to improve absorber dispersion would be beneficial.

In Fig. 8(b), the absorbance curve of the multi-layered skin phantom with the 4-mg/g coffee layer exceeds that of the phantom with the 8  mg/g coffee layer for the 580- to 700-nm wavelength range, and in Fig. 8(c), the reflectance curve of the skin phantom with the 4-mg/g coffee layer falls under that of the phantom with the 8-mg/g coffee layer in the same spectral range. These effects are attributed to slight differences in the layer thickness and local inhomogeneity, which are accounted for in the recovered absorption coefficient [Fig. 8(d)].

The reference absorption coefficient shown in Fig. 8(d)^51^ corresponds to the Caucasian skin, which explains the closest agreement with the layer containing 2-mg/g coffee. In the assembled multi-layer phantoms, the presence of the dermal and fat layers substantially increases overall scattering.

For further skin tone representation analysis, ITAs were calculated for the multi-layered phantoms with an epidermis-mimicking coffee-containing layer on top, using the equation proposed by Chardon et al.^52^

$$\mathrm{ITA}=(\frac{\arctan\left(L^{*}-50\right)}{b^{*}})\cdot \frac{180}{\pi },$$

where $L^{*}$ represents the luminance ranging from black (0) to white (100) and $b^{*}$ ranging from yellow to blue. To obtain the parameters $L^{*}$ and $b^{*}$, reflectance data within the visible wavelength range (400 to 700 nm), recorded with the spectrometer with an integrating sphere, were converted to CIE XYZ color space with MATLAB functions, using the CIE 1931 2 deg standard observer color matching functions^53^ and the CIE D65 standard illuminant.^54^ The XYZ values were subsequently transformed into CIELAB coordinates ($L^{*}$, $a^{*}$, and $b^{*}$), from which the ITA values were then calculated. The ITA values according to two classifications (older^52^ and recent, suggested in Ref. 55) for the fabricated phantoms (without and with blood-mimicking liquid) and the reference skin types from Ref. 48 are presented in Table 1.

**Table 1:** ITA-based skin tone classification of multi-layered skin phantom with coffee-containing layers.

Phantom/skin type	ITA (deg)	ITA classification^52^	ITA classification^55^
Phantom, 2-mg/g coffee	51.4	Light	Light
Phantom, 4-mg/g coffee	24.3	Tan	Medium
Phantom, 8-mg/g coffee	16.0	Tan	Medium
European^48^	47.7	Light	Light
South Asian^48^	27.7	Tan	Medium
African^48^	−5.6	Brown	Medium
Phantom + “blood,” 2-mg/g coffee	50.7	Light	Light
Phantom + “blood,” 4-mg/g coffee	18.7	Tan	Medium
Phantom + “blood,” 8-mg/g coffee	−7.5	Brown	Medium

The calculated ITA values indicate that the coffee-based phantoms reproduce a subset of human skin tones, corresponding primarily to lighter-to-medium skin reported in clinical datasets and correlating well with the target skin types in Ref. 48. Even though the phantoms do not cover the full ITA range observed across diverse human populations, which extends to substantially lower values (−70  deg and beyond) for much darker skin types,^55^^,^^56^ these results suggest that coffee can be used to reproducibly tune the coloration of silicone phantoms to represent multiple clinically relevant skin tone categories.

### 3.2 Vascular Structure, Micropump System, and Blood-Mimicking Liquid Perfusion

**Fig. 9:**
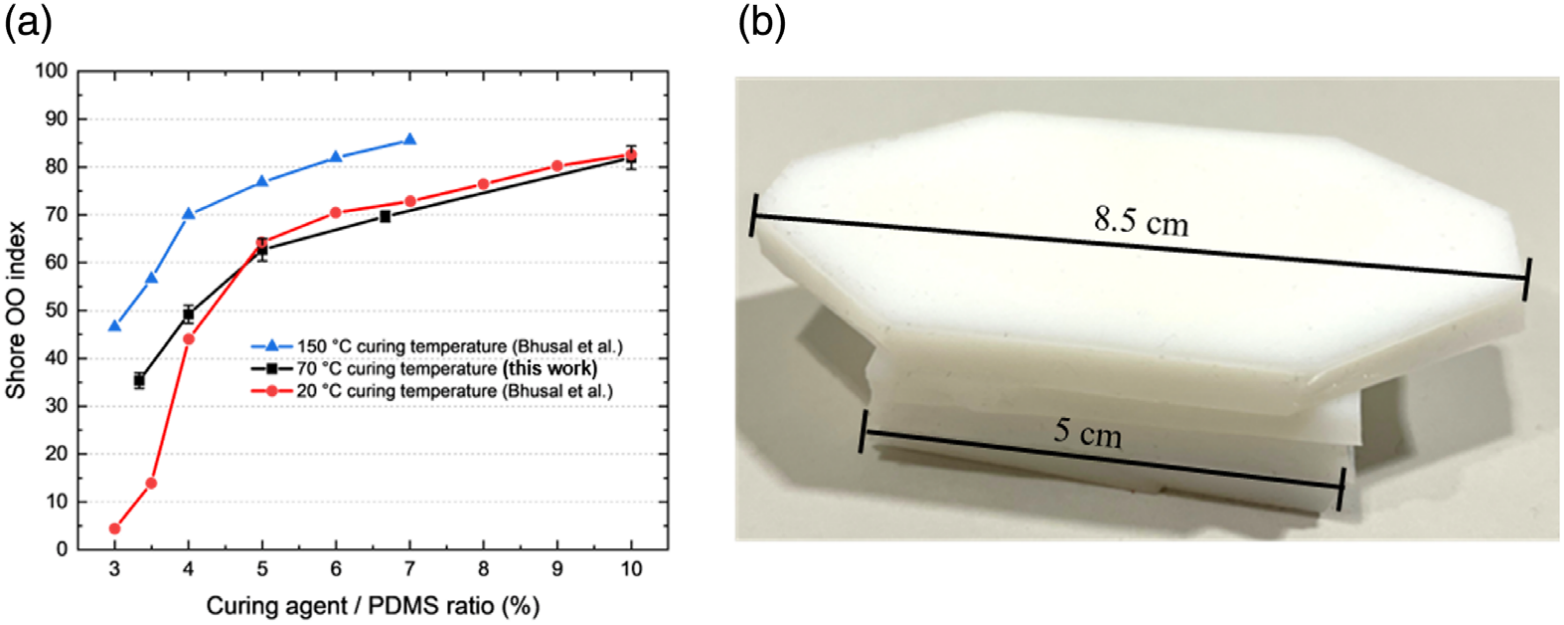
(a) Stiffness (Shore OO scale) of the phantom material depending on the curing agent/PDMS ratio cured at 70°C, compared with literature data for phantoms cured at 20°C (the red filled circles and the red curve) and 150°C (the blue triangles and the blue curve).^57^ (b) Multi-layered phantom (without coffee-containing epidermal layer), with the dimensions from Fig. 3 with the vascular structure (not visible) implemented between 1-mm-thick dermal and 20-mm-thick subcutaneous fat layers.

The vascular structure was implemented in the phantoms to enable blood-mimicking liquid perfusion. Vessel sizes of the structure were confirmed with optical coherence tomography imaging (Sec. S4 in the Supplementary Material). Mechanical properties of the phantom material were measured with a Shore OO durometer (AS-120OO, ATO, Diamond Bar, California, United States). Figure 9 shows the mechanical stiffness of phantoms with different curing agent/PDMS ratios compared with the work of Bhusal et al.^57^ and the structure of the fabricated multi-layered phantom (without coffee-containing layer).

Stiffness measurements of different curing agent/PDMS ratios show a logarithmic increase with an increasing curing agent amount at the curing temperature of 70°C. The data fall in-between phantoms cured at temperatures of 20°C and 150°C.^57^ The Shore index of skin, of around 30,^24^ corresponds to the lowest ratio of 3.3% (1:30). The second material (Ecoflex 00-30) also exhibited close-to-reality mechanical properties. However, PDMS was used for multi-layered phantoms due to its well-known tissue-like mechanical properties. In addition, PDMS is more optically transparent and has lower intrinsic absorption.

**Fig. 10:**
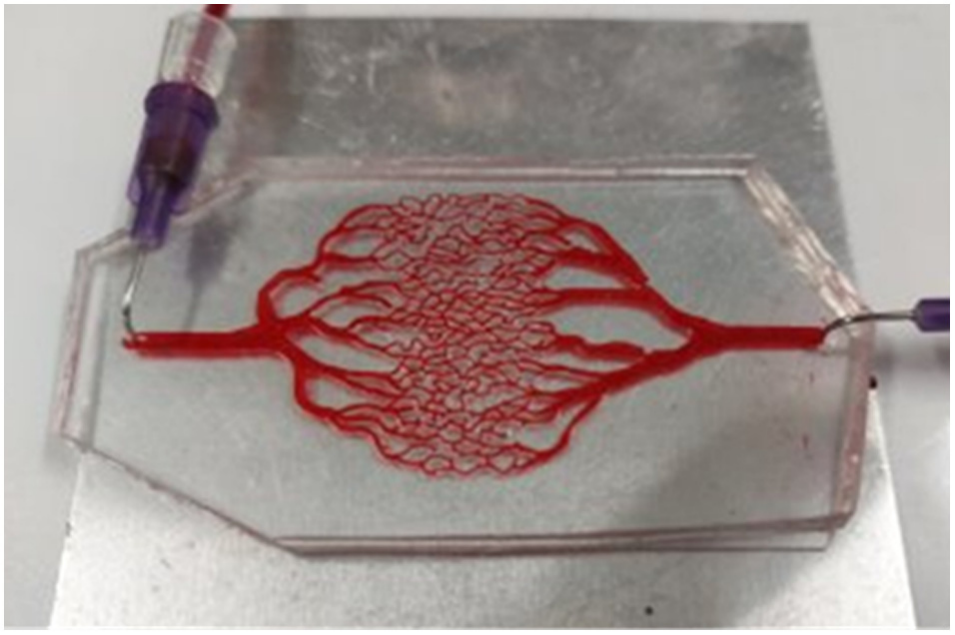
Perfusion of the blood-mimicking liquid through a transparent microfluidic phantom integrated into the micropump system.

When integrated into the micropump system, all phantoms with varying stiffness values enabled perfusion of blood-mimicking liquid under steady flow conditions. Figure 10 shows the liquid perfusion through a clear phantom (for illustrative purposes).

### 3.3 Hyperspectral Imaging

**Fig. 11:**
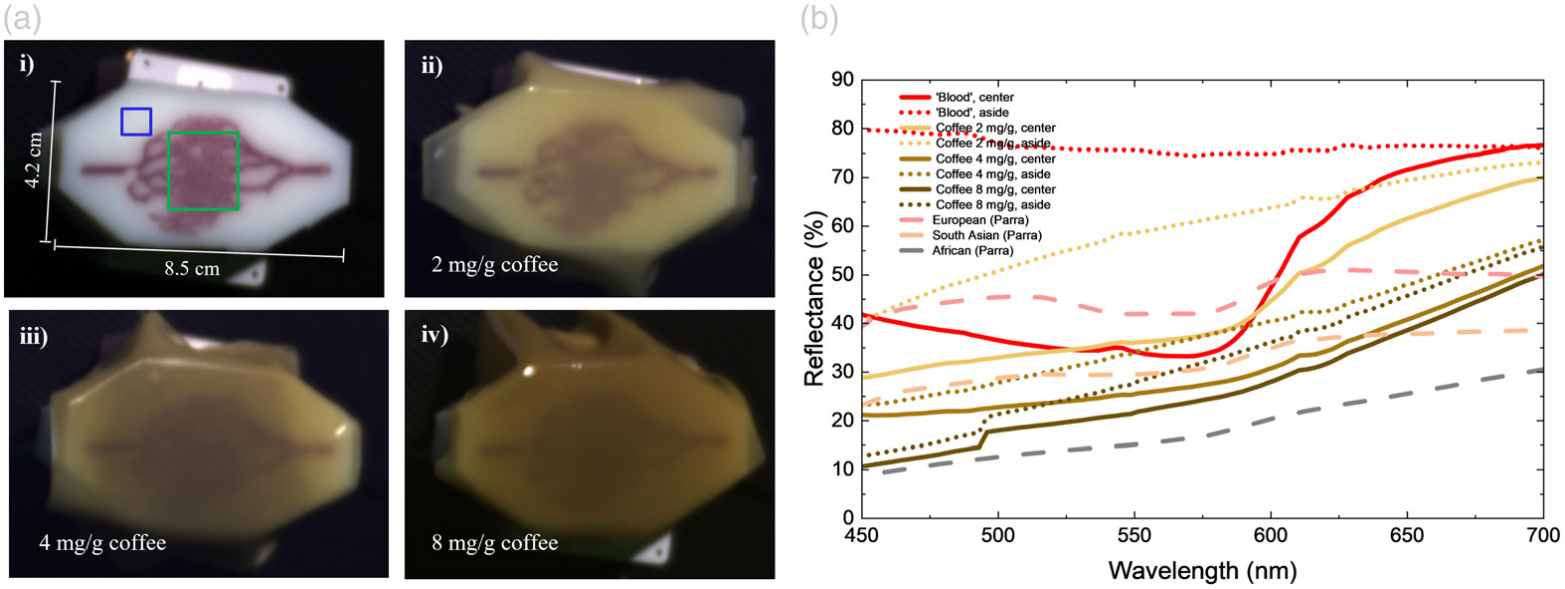
(a) Multi-layered phantom imaged by the HS camera (an RGB image): (i) perfused, with two selected ROIs denoted by the large green (“center”) and the small blue (“aside”) rectangles; (ii)–(iv) perfused and topped with a 2-mg/g (ii), 4-mg/g (iii), and 8-mg/g (iv) coffee-containing epidermal phantom layer, respectively. (b) Reflectance of perfused and non-perfused multi-layered phantoms, topped with different skin-tone epidermal layers in comparison to skin reflectance from Ref. 48.

HSI was used to detect the masking effect of the epidermal layer when imaging blood-mimicking liquid perfusion through the multi-layered phantom of different skin tones. Figure 11 shows phantoms imaged by the camera (RGB view) and the measured reflectance spectra of perfused and non-perfused phantoms.

Figure 11(a) shows the photos of the multi-layered skin phantom with the capillary structure, with blood-mimicking liquid. All coffee-containing epidermal layers (with coffee concentrations of 2 to 8  mg/g) are shown. Figure 11(b) presents the reflectance spectra of the phantom in each situation, from the area with capillaries (“center”) and without them (“aside”). The reflectance curves of the coffee-containing phantoms show decreased reflectance and the effect of the blood-mimicking liquid (very pronounced within the 450- to 600-nm spectral range). The darkest epidermal layer (8  mg/g coffee concentration) effectively masks “the blood”: the reflectance from the capillary area and the surroundings become hardly distinguishable. For comparison, the reflectance spectra for different targeted skin types (European, South Asian, and African) from Ref. 48 are also shown.

### 3.4 Stability of the Phantoms

**Fig. 12:**
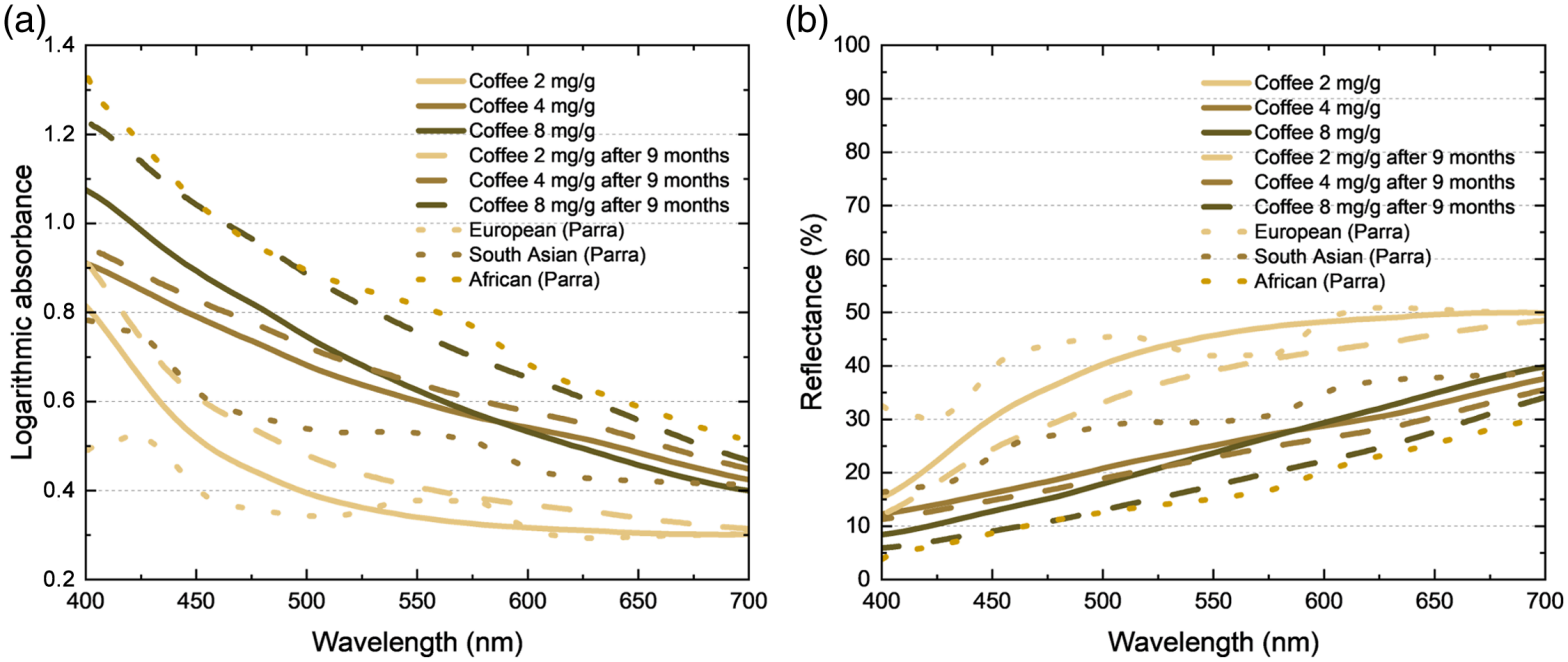
Comparison of absorbance (a) and reflectance (b) of the multi-layered phantoms immediately after and 9 months after fabrication, with reference values from Ref. 48.

To study the stability of the phantoms, the optical properties of the phantoms were remeasured 9 months after their fabrication. Figure 12 presents the initial measurements compared with the remeasurements.

After 9 months, absorbance of the phantoms increased slightly, 0.03 to 0.1 at 550-nm wavelength, while reflectance decreased by 2% to 6% at the same wavelength, for all phantoms. These consistent trends indicate modest time-dependent optical darkening, likely associated with diffusion of absorbing particles into the silicone matrix. The observed changes are favorable toward the implementation of darker skin tones.

## 4 Discussion

In this study, we developed optical phantoms mimicking the skin of different tones, with incorporated vascular structures and simulated blood flow. ZnO was validated as a suitable scattering agent, with concentrations optimized for multi-layered skin phantoms. Both commercial skin-tone silicone color pigments and coffee were evaluated as absorbers, with the former offering color uniformity and coffee providing optical spectra closely aligned with those of melanin. Commercial pigments are designed for the human eye, while our aim was quantitative spectral accuracy as measured by high-resolution spectrophotometry. Achieving a homogeneous dispersion of coffee within the silicone matrix is challenging due to particle clustering and requires further refinement. The slight color variations are not critical for the overall optical performance, as the multilayered structure averages out local differences, similar to the natural skin, which exhibits inherent pigment variability. Nevertheless, improving dispersion (e.g., by substituting water with a thinning liquid) would enhance the reproducibility of the phantoms. In terms of covering a broad range of skin tones, we have achieved a good match with the target skin types from Ref. 48, especially with the addition of the blood-mimicking liquid spanning from 51.4 to −7.5  deg in terms of the ITA metric. We admit, however, that the natural diversity of skin tones is significantly larger, reaching much lower ITA values, −70  deg and beyond) for very dark skins. Our preliminary steps in this direction indicate that implementation of higher coffee content (up to tens of mg/g) is feasible, thus making ITA value −70  deg fully implementable. Inclusion of other absorbers, such as synthetic melanin, could also be researched, although the incompatibility of an organic substance and the inorganic silicone matrix poses a new challenge.

The integration of the vascular structure and the micropump (artificial cardiovascular) system supports the utility of the phantom for simulating blood perfusion. The HSI analysis confirms the detectability of the blood-mimicking liquid through the implemented skin tones. The reflectance spectra showed decreased values in the blue-green part of the spectrum (associated with the absorption of the blood-mimicking liquid) from perfused regions, validating the ability of the phantoms to mimic close-to-reality optical changes. However, in the darker-toned phantoms, the difference in reflectance between perfused and non-perfused areas was markedly reduced. This illustrates the masking effect of the top-most epidermal layer containing coffee, in our case (and melanin in real skin), and highlights the challenge of detecting subsurface features through highly pigmented phantoms (and skin). Comparison with reference literature data for human skin reflectance indicates that our approach reasonably well reproduces reference skin types (European, South Asian, and African) but needs further refinement for covering very dark skin (as highlighted above).

Another limitation of the current phantom design is the static nature of the skin tone layers, which does not account for dynamic physiological variations, mainly oxygen saturation changes in the blood. This implies that in our future work, blood perfusion with controlled oxygenation through the phantoms should be implemented. Furthermore, comparison of phantom performance with real human skin using HSI is envisaged—aiming for recovery of skin physiological parameters.^58^^–^^60^ Overall, our results validated the potential of the fabricated phantoms as a practical tool for reducing racial bias in optical diagnostics while identifying clear pathways for further refinement.

## 5 Conclusion

This study presents the development of single- and multi-layered optical skin-mimicking phantoms across a range of skin tones (European, South Asian, and African), integrated vascular structures, and simulated blood flow to support the evaluation of optical medical and consumer wearable devices. The combination of ZnO-based scattering and coffee-based absorption enabled modeling of realistic skin optics, with superiority over the commercial silicone skin-tone pigments, while an artificial cardiovascular system allowed simulation of blood flow. The spectral signature of the blood-mimicking liquid was effectively masked by the darker superficial coffee-containing layer, which mimicked pigmented epidermis, thus reflecting real-world diagnostic challenges.

These findings emphasize the need for continuing the work for developing representative phantoms to implement more inclusive and accurate optical medical and consumer technologies. Future work will aim to further refine the optical properties of the phantoms for closer alignment with darker skin, to incorporate actual blood perfusion, and to validate performance through comparison with *in vivo* measurements of skin using a medical-grade HSI system.

## Supplementary Material

10.1117/1.JBO.31.7.075001.s01

## Data Availability

The data supporting the findings of this article are not publicly available due to internal corporate policy.
