# Supplementary material for: Enhancing skin tone representation in optical vascular phantoms
Source: J Biomed Opt. 2026 Jul 22;31(7):075001. doi: 10.1117/1.JBO.31.7.075001 (PMC13389507; doi:10.1117/1.JBO.31.7.075001)
Supplement: Supplementary file 1 [file JBO_031_075001_SD001.pdf]

Supplementary Material

Enhancing skin tone representation in optical vascular phantoms

Anni Ranta-Lassila,* Lauri Rannaste, Jarno Petäjä, Marko Korkalainen, Markku Alamäki, and Alexey Popov[1]

VTT Technical Research Centre of Finland, Kaitoväylä 1, Oulu, 90590 Finland

*Anni Ranta-Lassila, e-mail: anni.ranta-lassila@vtt.fi; [1]Alexey Popov, e-mail: alexey.popov@vtt.fi

1 Vascular structure

Human skin consists of three primary layers, the epidermis, dermis, and hypodermis, each differing in structure and function. The epidermis is the outermost layer, about 0.1 mm (100 μm) thick, and contains no blood vessels. Beneath it, the dermis ranges in thickness from approximately 0.4 mm to 4 mm depending on body site and is subdivided into the papillary dermis and the reticular dermis [61-68]. Below the dermis lies the hypodermis, composed predominantly of adipose tissue and containing larger nerves and blood vessels. Within the dermis, blood vessels form a dense and highly organized network composed of arterioles, venules, and capillaries. These vessels are arranged into two major horizontal plexuses: a superficial vascular plexus located near the boundary between the papillary and reticular dermis, and a deep vascular plexus positioned at the interface between the reticular dermis and hypodermis. Ascending arterioles and descending venules link these plexuses, while capillary loops extend upward into the dermal papillae. Capillaries are the smallest blood vessels in the dermis, measuring approximately 4–6 μm in diameter. Capillary loops in the papillary dermis widen to 17–22 μm before narrowing into true capillaries [45]. Arterioles in the papillary dermis also measure 17–22 μm, while postcapillary venules fall within a 10–15 μm diameter range [45]. Overall, vessel diameters in the dermis vary

widely, from as small as 7.5 μm to as large as 300 μm, depending on vessel type and depth [46]. Capillaries appear at multiple depths: 70 μm in the papillary dermis, around 300 μm in the upper dermis, and 1000 μm at the dermal-subcutaneous boundary [43].

2 Backside lithography

With backside lithography, it is possible to achieve more rounded capillary structure molds compared to the standard top-side lithography [47]. Another benefit is that the vascular height is proportional to the local width of the capillaries [47], thus, the same resist thickness will inherently lead to different heights of the capillaries if their width varies over the structure. It is notable that if the height of the resist layer is thin compared to the width of the capillary, it will result in flattened top and bottom parts of the vessels, with only walls retaining round shapes. The variables to achieve rounded shape are the diffuser, photoresist, used wavelength, exposure dose, resist thickness and capillary widths.

The mask layout (Fig. S1) consisted of geometrically identical vascular-like structures subjected to different scaling factors, with vascular dimensions of each pattern listed in Table S1.

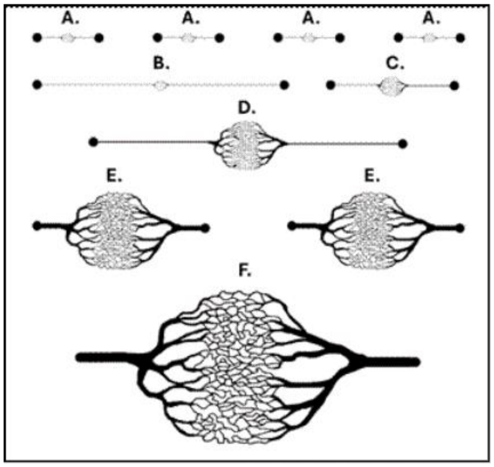

Figure S1. The mask layout.

Table S1. Vascular pattern dimensions.

Pattern	Pattern height [µm]	Pattern width / length [mm]	Vascular width min/max [µm]
A	200	2.05 / 15.35	10 / 50
B	200	2.05 / 55.35	10 / 50
C	200	4.10 / 30.70	20 / 100
D	200	10.10 / 68.77	50 / 250
E	200	16.25 / 38.27	110 / 1100
F	200	32.50 / 74.54	236 / 2000

The backside lithography functionality in the mask aligner was achieved simply by positioning a diffuser plate (125 mm x 125 mm Opal diffuser #83-389, Edmund Optics) on top of the mask holder. Figure S2 presents the schematic configuration of the backside lithography in the mask aligner. The thickness of the mask was 1.5 mm.

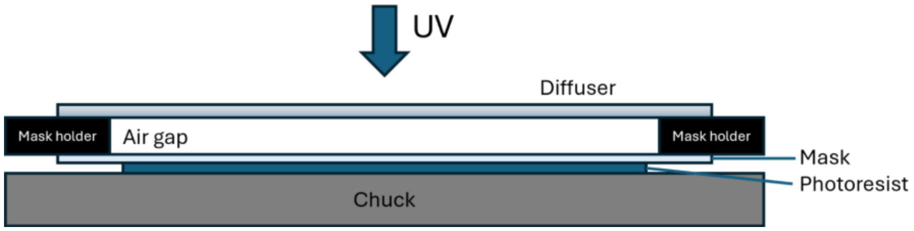

Figure S2. Schematic presentation of the backside lithography configuration.

Typically, dry films are deposited on the substrate before photolithography processing. To get the rounded shape via the backside lithography, the dry film should have been laminated on the mask. This would mean that each processed dry film would have consumed one mask, since the mask would then be an integral part of the mold. To avoid this inefficiency and reduce the manufacturing cost, we sought a method that would allow for mask reuse. Our solution was to

process the dry film without lamination, enabling the mask to be reused for multiple cycles. Otherwise, the processing was done according to the manufacturer's guidelines, resulted in individual vascular molds (Fig. S3). These molds were then glued on separate metal plates for casting and implementation of tubeless capillary strucutures. In this approach, the location and orientation of the vascular molds on the metal plates can be varied.

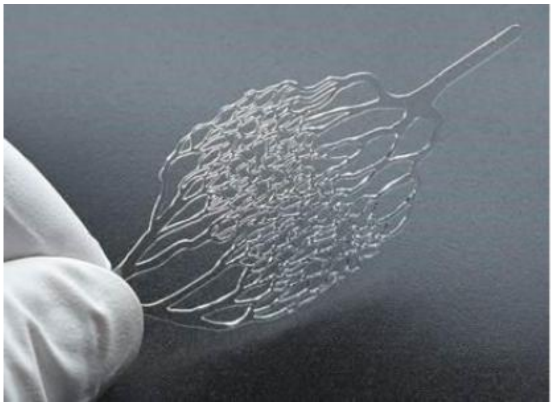

Figure S3. A vascular structure fabricated on dry film photoresist with UV backside lithography (pattern F).

3 Silicone pigment phantoms

Fig. S4 presents silicone pigment phantoms compared to the optical properties of real skin in Parra *et al.* (2007) [48].

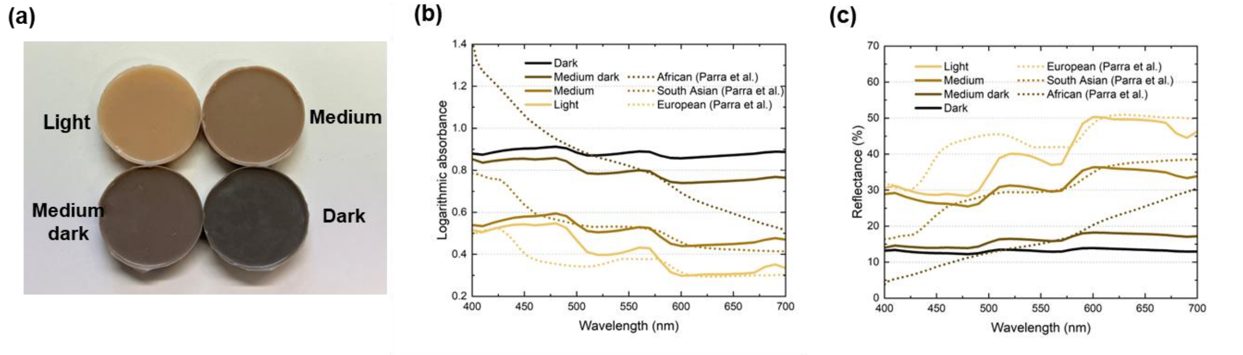

Figure S4. a) 2-cm thick single-layer homogeneous phantoms with silicone pigments and their b) absorbance and c) reflectance, compared to real skin data from [48].

4 Imaging of the microfluidic vessel structure

Imaging of the transparent fabricated microfluidic vessel structure was implemented using an optical coherence tomograph (OQ Labscope 3.0, Lumedica) operating at 840-nm wavelength, with an axial resolution of 8 µm in air and ca. 5.7 µm in transparent PDMS medium (RI = 1.407). To obtain necessary contrast between the capillaries and the surroundings, the structure was filled with water-suspended 500-nm polystyrene (PS) microspheres (59769, Sigma-Aldrich): 0.1 mL of stock solution (10% PS particles in water, as purchased) was diluted in 2 mL of DI water. The images (4x/0.1 objective) (Fig. S5) showing cross-sections of the capillaries indicate a varied vessel width of 200-2500 µm, with the height around 170 µm (defined by the thickness of the resist layer). Flattened bottom and top parts are visible, due to the reason described above.

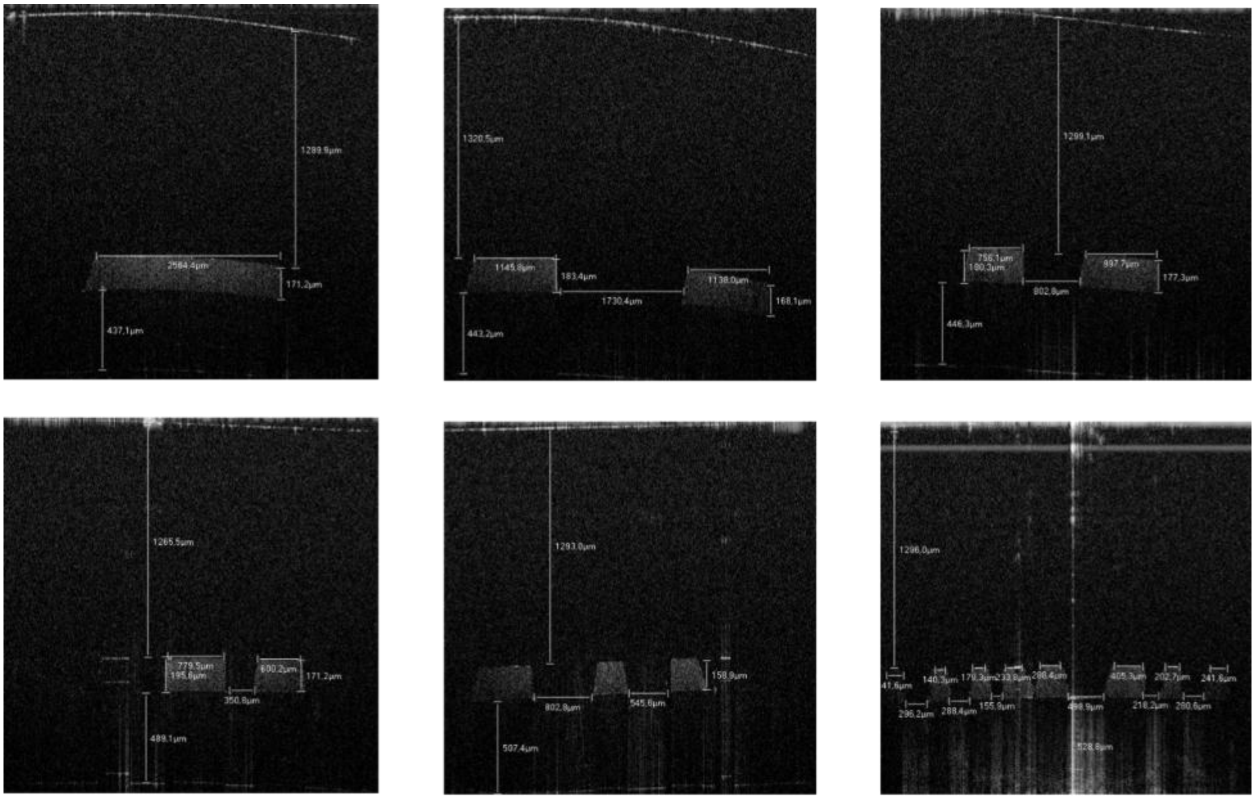

Figure S5. Optical coherence tomography (OCT) images of the fabricated microfluidic structure (cross-sections).
